# Curriculum Innovations: A Novel Neurology Clinician-Educator Program

**DOI:** 10.1212/NE9.0000000000200175

**Published:** 2024-11-27

**Authors:** Rafid Mustafa, Sherri A. Braksick, Jeremy K. Cutsforth-Gregory, David B. Burkholder, Eoin P. Flanagan, Jonathan Graff-Radford, Christopher J. Boes, Andrea N. Leep Hunderfund, Lyell K. Jones, Elizabeth A. Coon

**Affiliations:** From the Department of Neurology, Mayo Clinic, Rochester, MN.

## Abstract

**Background and Objectives:**

Neurology residents serve as frontline teachers for junior trainees but often lack formal training in medical education. We developed a novel longitudinal curriculum to enhance the teaching skills and educational leadership of residents interested in pursuing careers as clinician-educators.

**Methods and Curriculum Description:**

We developed and piloted a Neurology Clinician-Educator Program (NCEP) with the following goals: (1) improve resident satisfaction with opportunities to develop teaching skills, (2) improve resident satisfaction with opportunities to transition into a clinician-educator role after training, and (3) enhance resident teaching skills using evidence-based strategies. The NCEP was implemented on an optional basis for senior neurology residents at a single academic institution and applied through a thematic framework centered around experiential learning theory. The program involved 2 immersive 3-week experiences including both didactic and application formats. These sessions were integrated through the implementation of capstone education projects, facilitating the translation of theoretical knowledge acquired from the NCEP into real-world practice. Satisfaction with opportunities to develop clinician-educator skills was measured through preimplementation and postimplementation surveys distributed to the entire residency cohort while participant progression of educator skills was measured through faculty-guided self-assessment of the Accreditation Council for Graduate Medical Education (ACGME) Clinician Educator Milestones framework.

**Results and Assessment Data:**

Eight residents completed the 2-year NCEP in its initial iteration. Residents throughout the entire residency program (response rate 24/27 before implementation and 25/27 after implementation) reported significantly higher satisfaction with opportunities to develop teaching skills (64% after implementation vs 33% before implementation, *p* = 0.032) and with opportunities to transition into a clinician-educator role following training (68% after implementation vs 29% before implementation, *p* = 0.007) after implementation of the NCEP. Among the 8 residents who completed the NCEP, faculty-guided self-assessment improved significantly (*p* < 0.05) in 14 of 20 ACGME Clinician Educator Milestone domains.

**Discussion and Lessons Learned:**

This study demonstrates the successful implementation of a novel curriculum for neurology residents interested in becoming clinician-educators. The NCEP was well received by participants, resulted in improved satisfaction with opportunities to develop clinician-educator skills, increased learning of education concepts and skills, and resulted in behavioral change reflected in various capstone education projects.

## Introduction

Many neurologists are interested in pursuing academic careers as clinician-educators, who combine clinical practice with teaching, curriculum development, educational research, and/or leadership roles. Recent surveys of both neurology and non-neurology trainees indicate that most intend to pursue careers in academic medicine, with most aspiring to become clinician-educators.^1,2^ Yet, for decades, clinician-educators have faced challenges in academia compared with their more traditional clinician-scientist colleagues.^3^ More recent recognition of success in medical education careers has allowed for neurologists to better advocate for funding, more easily achieve advancement and promotion, engage in educational scholarship, and attain leadership roles at national and international levels.^4-8^ While education remains core to the future of neurology and interest and opportunities for educators continue to grow, training medical educators has often been a largely passive process with a lack of formal development and mentorship opportunities for aspiring neurology clinician-educators.

Residents, particularly senior residents, serve at the frontline of clinical teaching for junior trainees through roles in formal and informal didactics, with involvement in program leadership, and as role models to their peers. While up to one-fourth of a resident's time in training may be spent supervising or teaching junior trainees,^9^ the formal development of medical teaching skills is not an explicit requirement of neurology programs in the Accreditation Council for Graduate Medical Education (ACGME) requirements.^8^ Therefore, the development of teaching skills traditionally is a responsibility of the resident and the program. Neurology training programs are beginning to offer longitudinal clinician-educator tracks in which educational experiences can be tailored to specific career goals. These may involve didactics, education electives, research opportunities, bedside teaching, and mentored projects.^8^ Formal medical education curricula or postgraduate fellowships in education in specialties such as internal medicine or emergency medicine have been shown to significantly improve teaching abilities, reinforce trainee knowledge, and strengthen postresidency career opportunities in medical education.^9-11^ Within neurology, however, only 1 specific education fellowship exists today, the American Academy of Neurology Institute Medical Educator Fellowship to support medically oriented education research.^8^

A Neurology Clinician-Educator Program (NCEP) was developed for trainees in the Mayo Clinic Neurology Residency to address this discrepancy between increasing clinician-educator phenotype interest among trainees in neurology and the limited availability of formal clinician-educator training programs. The foundational framework for this curriculum was informed by themes of the Harvard Macy Institute (HMI) Program for Educators in Health Professions and experiential learning theory.^12-14^ Experiential learning theory helps advance the skills of medical educators by understanding learning through experience and learning through reflection of experience and importantly guiding educational planning by understanding how different physicians learn within the continuing medical education domain. These frameworks led to our curriculum's longitudinal and highly experiential nature.

## Problem Statement

Interest in medical education as a long-term career path has seen rapid growth, and a growing number of neurology trainees are interested in pursuing a career as a clinician-educator. Despite this trend, formal opportunities for training of clinician-educators in neurology are lacking. Other medical subspecialties have been successful at developing residency-integrated programs focused on teaching in addition to postgraduate education-based fellowships. These programs have been shown to significantly improve trainee teaching abilities, reinforce trainee knowledge, and improve post-training career opportunities focused on education. Yet, such programs are limited or in their infancies within neurology. In this study, we examine a longitudinal and experiential curriculum to help develop neurology clinician-educators. We aim to determine whether this approach is feasible and effective to training neurology residents in medical education.

## Objectives

We developed and piloted a NCEP at Mayo Clinic with the following goals:

1. Improve neurology resident satisfaction with opportunities to develop their teaching skills.
2. Improve neurology resident satisfaction with opportunities and mentorship to transition into a clinician-educator role after training.
3. Enhance neurology resident teaching skills with a focus on evidence-based teaching strategies.

## Methods and Curriculum Description

### Curriculum Participants

The curriculum was implemented for all senior residents in the Mayo Clinic Adult Neurology Residency Program, on an optional basis. Postgraduation year (PGY) 3 and 4 residents were invited to participate.

### Curriculum Structure and Content

The NCEP was designed with elements to enhance transformational learning, allowing learners to develop new perspectives about one's self and work and changing one's actions in light of that new perspective.^12,13,15^ This was applied through 5 interconnected themes (Figure, panel A): Examining Assumptions; Teaching and Learning Theory; Curriculum Design; Providing and Receiving Feedback; and Leadership, Scholarship, and Innovations. Specifically, the program involved an immersive 3-week experience in the winter of the residents' PGYs 3 and 4, allowing for an alternating complement of 3 themes each year (Figure, panel B). The theme of Examining Assumptions, centered around David Kolb's experiential learning theory,^13^ served as the foundation on which the remainder of the curriculum was built.

**Figure:**
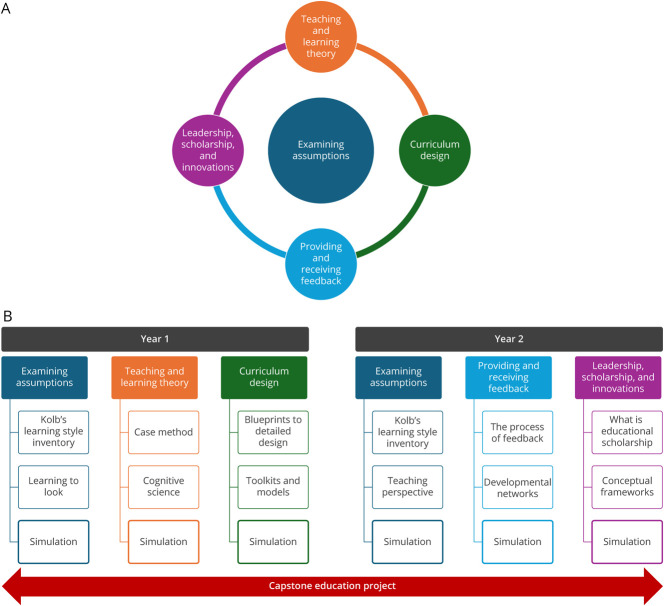
Neurology Clinician-Educator Program Overview Neurology clinician-educator program themes (A) and structure (B).

Formats included interactive group didactic sessions and exercises, problem-based learning, case-method teaching, observations, and small and large group discussions. Modeled from the HMI Program for Educators in Health Professions, observational reflection of shared experiences was used to expose individual assumptions about learning, allowing participants to step back and examine experiences with new perspectives. Participants worked with their colleagues to construct individualized educational principles that would shape their future approaches to teaching.^12^ The individual 3-week immersive experiences were tied together through capstone projects, in which participants pursued an education project matching their individual interests and addressing an educational need within the neurology residency program. Development of these projects was influenced through regular faculty and peer group consultation, with key exploration of project goals, program needs, stakeholder input, consideration of resources needed for execution as well as potential barriers, and methods for project evaluation. In essence, the projects functioned as an avenue to translate theory learned from the NCEP into real-world practice.

### Curriculum Implementation

The NCEP was implemented in 3-week sessions, each in the winter of 2022 and 2023, in addition to quarterly touch points for ongoing project consultations. The immersive sessions were scheduled during residents' slated didactic time and used both in-person and video conference formats. Each week of the program focused on 1 particular core theme, with 3 individual 1-hour immersive sessions each week (Figure, panel B). The first 2 sessions within a week were conducted predominantly in interactive didactic formats while the last session within each week involved simulation or application-based activities. Most of these sessions required the residents to complete approximately 1–2 hours of presession and/or postsession course work outside of the classroom environment. A detailed curriculum outline of the NCEP can be found in the supplementary materials (eAppendix 1).

As an example, the focus of Year 1 Week 1 of the NCEP was on the theme of Examining Assumptions (Figure, panel B). Throughout this week, participants developed their skills in understanding differences between various learning and teaching styles, so that they could more expertly use their strengths and better implement the most effective teaching style for unique educational scenarios. Before Session 1, all participants completed Kolb's Learning Style Inventory.^13^ Then, the in-person session was used to discuss Kolb's approach to experiential learning, explore each individual participant's preferred learning style, and strategize how to be an effective educator when working with learners whose preferred learning styles differ from one's own. Session 2 delved deeper into implicit biases, introduced the ladder of inference as a tool to reduce implicit bias, and explored strategies for intentional design of educational programs and learning environments that are principle driven.^16^ As a postsession assignment, participants were required to observe a teaching session of their choice in the classroom or on the wards and methodically record observations, reactions, and assumptions that developed in their observation of the learning environment to walk away with thoughtful questions to help them grow as educators. In Session 3, these concepts were all put into application through a microteaching exercise, where participants each delivered a very brief teaching session on a topic of their interest to a small group of peer and faculty colleagues. At the conclusion of the teaching session, the presenter stepped back while the remainder of the small group gathered to develop reinforcing and constructive feedback. The presenter then returned and was provided this feedback by their peer colleagues, with the ultimate goal that all participants grow in examining experiences such as this with new perspectives.

### Curriculum Assessment

Outcome measures for the NCEP were tied directly to the original objectives. Preimplementation and postimplementation surveys were distributed to the entire Mayo Clinic Adult Neurology Residency Program cohort to evaluate neurology resident satisfaction with opportunities to develop their teaching skills, resident satisfaction with opportunities and mentorship to transition into a clinician-educator role after training, and interest in a future clinician-educator career. Satisfaction was measured as a binary outcome, requesting trainees to simply respond whether they were fully satisfied or unsatisfied with the respective opportunities available. All residents in the residency program were surveyed, instead of solely those who participated in the NCEP, to assess the larger impact of the NCEP on perceptions of professional development opportunities for medical education among all residents.

To measure participant learning (Kirkpatrick level 2),^17^ we used the ACGME Clinician Educator Milestones framework.^18^ This framework was launched in 2022 by the ACGME in collaboration with the Accreditation Council of Continuing Medical Education, Association of American Medical Colleges, and American Association of Colleges of Osteopathic Medicine, providing a comprehensive list of fundamental skills and responsibilities for teaching faculty, particularly those in medical education leadership. The individual clinician-educator milestones are categorized into 5 thematic competencies (Universal Pillars; Educational Theory and Practice; Well-Being; Diversity, Equity, and Inclusion in the Learning Environment; and Administration) and developmentally organized based on the Dreyfus model to illustrate 5 levels of faculty achievement in medical education: level 1—novice; level 2—advanced beginner; level 3—competent learner; level 4—proficient learner; and level 5—expert.^19^ Following recommendations from the Society of General Internal Medicine Education Committee to apply these clinician-educator milestones, we used faculty-guided self-assessment before implementation and after implementation of the NCEP to assess milestone achievement and reflect on areas for continued professional growth.^20^ This individual assessment was guided by the faculty course director who was aware of the intervention.

### Statistical Analyses

All statistical analyses were completed using R version 4.0.3 (R Foundation for Statistical Computing, Vienna, Austria). Unpaired categorical data were compared using Fisher exact tests while paired ordinal data were compared using paired Wilcoxon signed-rank tests.

### Standard Protocols Approvals, Registrations, and Participant Consents

This educational program met Mayo Clinic's criteria for certification as a quality improvement project rather than human subject research and was exempt from institutional board review.

### Data Availability

Anonymized data not published within this article will be made available to qualified investigators on request.

## Results and Assessment Data

The entire Mayo Clinic Adult Neurology Residency Program cohort (n = 27) was surveyed to evaluate resident satisfaction with opportunities to develop clinician-educator skills before (2021) and after (2024) implementation of the NCEP. The response rate was 24/27 for the preimplementation cohort and 25/27 for the postimplementation cohort. Overall, residents throughout the program reported significantly higher satisfaction in their opportunities to develop teaching skills and to transition into a clinician-educator role following training after implementation of the NCEP (Table 1).

**Table 1:** Resident Satisfaction With Opportunities for Clinician-Educator Professional Development Before and After Implementation of the Neurology Clinician-Educator Program

	Before intervention (N = 24), n (%)	After intervention (N = 25), n (%)	*p* Value^a^
Satisfaction of opportunities to develop teaching skills	8 (33.3)	16 (64)	0.032
Satisfaction of opportunities and mentorship to transition into a clinician-educator role after training	7 (29.2)	17 (68)	0.007

a Fisher exact test.

The NCEP was completed by 8 neurology residents between 2022 and 2023. Faculty-guided self-assessment of the ACGME Clinician Educator Milestones for these 8 residents before and after completion of the NCEP is provided in Table 2. The residents improved significantly in most of the milestone domains, with trends toward improvement in the remaining milestones of well-being, professionalism in the learning environment, learner assessment, feedback, and leadership skills. After completion of the NCEP, all 8 residents achieved a level of 3 (competent learner) or higher among all 20 clinician-educator milestones.

**Table 2:** Faculty-Guided Resident ACGME Clinician Educator Milestone Assessment Before and After Implementation of the Neurology Clinician-Educator Program

	Before intervention (N = 8) Median (IQR)	After intervention (N = 8) Median (IQR)	*p* Value^a^
Universal pillars			
Reflective practice and commitment to personal growth	3 (3–3.25)	4 (4–4)	0.026
Well-being	3 (2–3.25)	3 (3–4)	0.24
Recognition and mitigation of bias	2.5 (2–3)	4 (4–4)	0.019
Commitment to professional responsibilities	3 (3–3.25)	5 (4–5)	0.034
Educational theory and practice			
Teaching and faculty learning	2.5 (2–3)	4 (4–4)	0.012
Professionalism in the learning environment	2.5 (2–4)	3.5 (3–4)	0.17
Learner assessment	2 (1.75–3)	3 (3–3)	0.053
Feedback	2.5 (1.75–3)	3.5 (3–4)	0.057
Performance improvement and remediation	2 (1.75–2)	3.5 (3–4)	0.01
Programmatic evaluation	1 (1–2)	3 (3–3.25)	0.018
Learner professional development	1 (1–2.25)	3 (3–3.25)	0.031
Science of learning	2 (0.75–2.25)	4 (3–4)	0.022
Medical education scholarship	0 (0–1)	3 (3–3.25)	0.012
Learning environment	2 (1–2.25)	4 (3–4)	0.026
Curriculum	1 (0.75–2)	4 (3–4)	0.013
Well-being			
Well-being of learners and colleagues	2.5 (1.75–3)	3 (3–3.25)	0.053
Diversity, equity, and inclusion in the learning environment	2 (2–3)	3 (3–4)	0.048
Administration			
Administration skills	1.5 (0–2.25)	4 (3.75–4)	0.035
Leadership skills	2.5 (2–3)	3 (3–4)	0.19
Change management	1 (0–3)	4 (3–4)	0.02

Abbreviations: ACGME = Accreditation Council for Graduate Medical Education; IQR = interquartile range.

a Paired Wilcoxon signed-rank test.

The capstone education projects of the 8 residents who completed the NCEP are presented in Table 3. Two of these 8 projects have gone on to receive educational grant funding at the institutional level.

**Table 3:** Capstone Education Projects of the Neurology Clinician-Educator Program

Project title	Objectives	Assessment data	Target population	Educational focus	Progress to date
A neurointerventional pathway interest group for neurologists	1. Provide guidance and mentorship for neurologists applying into interventional neurology 2. Establish connections with neuroendovascular specialists at Mayo Clinic and other institutions 3. Discuss topics of interest in interventional neurology	1. Member feedback on the effectiveness and usefulness of this interest group 2. Objective measures will include the number of successful applications into the field of interventional neurology from the members	Neurology residents	Mentorship and guidance in the field of interventional neurology	• Proposal developed and submitted • Mentorship connections and pipeline established • First interest group meeting to take place in coming months
Designing and implementing a resident-focused movement disorders curriculum	1. Introduce residents to the different movement phenomenologies and the common and/or important movement disorders 2. Provide residents a structured framework to identify different phenomenologies 3. Improve resident subjective comfort in recognizing and managing movement disorders	There will be 3 formal assessments—1 before the course, 1 at the half-mark, and 1 at the end of the curriculum. These will consist of 5 brief videos of different phenomenologies. Residents will be asked to (1) identify the phenomenology and (2) describe the movement (as if describing to a colleague). There will also be a precourse and postcourse survey regarding resident comfort with different phenomenologies and movement disorders as a whole Short-term indicators for progress: Improvement in the half-mark assessment compared with the preassessment Outcomes to define success: 1. Improved ability to recognize different movement phenomenologies (objective) 2. Improved resident comfort with movement disorders (subjective)	Neurology residents	Teaching and education; curriculum design	• Proposal developed • Pilot lectures trialed • Awaiting formal approval into residency curriculum
Development of an application for integration of peripheral nervous system localization and electromyography study design	1. Improve medical student and resident skills in peripheral nervous system localization 2. Improve neurology resident and physical medicine and rehabilitation resident skills in planning electrophysiologic studies	Short-term indicators for progress: 1. Pre-Likert scale and post-Likert scale self-evaluations for medical students and residents regarding their familiarity with peripheral nervous system localization 2. Pre-Likert scale and post-Likert scale self-evaluations for neurology residents and physical medicine and rehabilitation residents regarding their familiarity with planning of electromyography studies Outcomes to define success: 1. Statistically significant improvement on the evaluations	Medical students and residents, including neurology and physical medicine and rehabilitation residents	Teaching and education; application design; clinical tools	• Needs assessment completed • In active consultation with software design experts
Development of an online interactive pediatric neurology curriculum for primary care providers	1. Improve the knowledge and confidence of primary care providers in managing common neurologic diseases 2. Enable effective triage in regions with high patient-to-provider ratios, directing cases requiring evaluation by pediatric neurology	A pre-post study design will assess the impact of the online learning module, conducting assessments before and after task completion. Interactive 15-min videos on selected topics will be designed, accompanied by an asynchronous self-learning component emphasizing knowledge and comprehension, followed by a synchronous small-group session focusing on applying knowledge to clinical cases Learners will undergo a precourse assessment before engaging in self-directed learning materials, followed by a postcourse assessment within a week of completing their modules. These assessments will gauge satisfaction with the pilot pediatric neurology curriculum, learners' confidence and knowledge in assessing cases with neurologic illnesses, and confidence in triaging patients	Primary care providers	Teaching and education; curriculum design	• Educational grant funding received • Curriculum outline completed • One-third of online interactive curriculum developed
Development of a resident curriculum for sex and gender in neurology	1. Increase trainee understanding of how common neurologic presentations can be considered through the lens of sex and gender 2. Build a working group of neurologists and other specialists (GIM, OB/GYN, primary care) as resources for curriculum development and clinical care consultations 3. Develop trainee understanding of sex and gender identity and inclusivity in the hospital and clinic 4. Build confidence to manage patients through the lens of sex and gender with an evidence-based approach	Short-term indicators for progress: 1. Prequizzes and postquizzes with cases based on lectures to be given regarding typical situations where sex and gender arise in neurologic conditions 2. Presurveys and postsurveys to participants regarding their confidence level Outcomes to define success: 1. Statistically significant differences in performance of the above 2. Increased interest in studying the role of sex and gender in neurologic disease by our trainees	Neurology residents	Clinical care; diversity, equity, and inclusion	• Educational grant funding received • Curriculum outline completed • Lecturers identified • Formal approval received to incorporate into residency curriculum • Official curriculum launch in coming months
Development of a resident-led case conference	1. Create a forum for neurology training to present clinical cases to their peers 2. Foster teaching opportunities between trainees in different postgraduate years	Short-term indicators for progress: 1. Attendance at the conference 2. Postgrad year distribution of conference attendees and presenters Outcomes to define success: 1. Higher percentage of trainee attendance at Case of the Week	Neurology residents and fellows	Teaching and education; curriculum design	• Proposal developed • Pilot conference trialed • Awaiting formal approval from residency program
Minimizing ICU anxiety—development of an ICU reference tool for trainees	1. Develop a quick reference handout/book for residents/trainees that focuses on management of disease processes and complications of disease processes 2. Reduce resident/trainee anxiety surrounding ICU rotations	1. Survey of resident experience leading up to ICU rotations before and after implementation 2. Collect data on points of stress that can be included in the handbook Short-term indicators for progress 1. Pre-Likert scales and post-Likert scales regarding resident anxiety leading up to and during ICU rotations Outcomes to define success: 1. Statistically significant improvement on scales noted above	Neurology residents	Teaching supplementation (i.e., to the ICU course already provided)	• Proposal developed • Reference handbook in development
Peripheral neuroanatomy and neuromuscular examination teaching modules	1. Create engaging online material to teach clinically relevant peripheral neuroanatomy to a variety of learners and improve mastery of the neuromuscular examination	1. Pre/post-neuroanatomy tests 2. Qualitative surveys 3. Faculty assessment of neuromuscular examination of trainees pre/post-videos	Medical students, neurology residents, and fellows	Teaching and education; curriculum design	• Curriculum completed, launched, and currently in use • Curriculum assessment in process

Abbreviation: ICU = intensive care unit.

## Discussions and Lessons Learned

This study provides evidence of the successful implementation of the Neurology Clinician-Educator Program for neurology residents as a professional development curriculum to address the gap between increasing clinician-educator career development interest among trainees in neurology and the limited availability of formal clinician-educator training programs. The primary impacts reported in this project focus on resident satisfaction with opportunities to develop their teaching skills and to transition into a clinician-educator role after training, as well as milestone-based achievement in various medical education domains. These results demonstrate preliminary findings of positive impact in both resident satisfaction with the aforementioned goals (Kirkpatrick level 1) and milestone-based achievement of learning (Kirkpatrick level 2).^17^

The NCEP was designed strategically using themes of the HMI Program for Educators in Health Professions, with a focus specifically on the learning environment of neurology trainees. We used the Program for Educators in Health Professions framework, given HMI's long history of clinician-educator development and previous demonstration of success with long-term changes in teaching behaviors, engagement in new educational endeavors, achievement of educational grant funding, and renewed identification as clinician-educators among graduates from the HMI program.^12^ Central to this model, as implemented in the NCEP, is Kolb's experiential learning theory, which allows for an immersive experience in a high-challenge, high-support environment where early-stage medical educators are able to better understand learning through experience and through reflection of experience.^13^

The experiential learning theory model of the NCEP—using immersive, interactive group sessions and exercises; problem-based learning; case-method teaching; individualized applications of learning; and development of new skills in exploring assumptions and principles—is successful in learner professional development, particularly in their growth of identity as medical educators. We evaluated resident satisfaction outcomes with preimplementation and postimplementation surveys distributed to the entire Mayo Clinic Adult Neurology Residency Program. The response rates were strong, over 88% in both cohorts, and the data collected suggest higher resident satisfaction with opportunities to develop teaching skills and to transition into a clinician-educator role following training after implementation of the NCEP.

Curricular outcomes were further evaluated with faculty-guided self-assessment of the ACGME Clinician Educator Milestones framework to assess changes in residents' learning in their growth as clinician-educators. Residents who completed the NCEP demonstrated significant improvement in milestone achievement in most of the domains, with most notable improvements in the domains of Recognition and Mitigation of Bias, Teaching and Faculty Learning, Performance Improvement and Remediation, Programmatic Evaluation, Medical Education Scholarship, Curriculum, Administration Skills, and Change Management. Several of these domains are strongly intertwined with Kolb's experiential learning theory addressed through the foundational theme of the NCEP, Examining Assumptions, while others were incorporated into the remaining themes of the NCEP: Teaching and Learning Theory; Curriculum Design; Providing and Receiving Feedback; and Leadership, Scholarship, and Innovations. There were trends toward improvement in the remaining milestones of well-being, professionalism in the learning environment, learner assessment, feedback, and leadership skills. The first 2 of these, where residents demonstrated the smallest degree of improvement after the NCEP, were not domains directly addressed by the course, potentially accounting for the limited degree of change.

The implementation of the NCEP demonstrates feasibility by the successful execution and retention of the program in the past 3 years. Despite its optional nature, the course was completed and well attended by 8 residents among a cohort of 18 eligible senior residents within the adult residency program at our institution. This likely coincides with the ongoing growth of interest in medical education careers in neurology and is a reflection of the genuine interest among the residents for professional development in medical education. Successful adoption of the program is supported by the increased satisfaction among residents with opportunities to develop clinician-educator skills and more importantly by the behavioral change reflected in their various capstone education projects they have initiated or completed.^21^

This study has several limitations. First, the satisfaction surveys completed before and after implementation of the NCEP could have been influenced by several confounders, such as changes in residency program leadership during the time of the study, influences from other curricula within the residency, proximity to the peak of the coronavirus disease 2019 pandemic between the presurvey and postsurvey cohort, or other intrinsic phenomena unique to each residency cohort. Nevertheless, we surveyed the entire adult neurology residency cohort at the preimplementation and postimplementation time points to minimize selection bias and ensure that the participants were not only those with strong interests in medical education. In addition, the response rates in both cohorts were strong. The strong response rate coupled with increased satisfaction with medical education professional development opportunities throughout the residency suggests a ripple effect throughout the residency program beyond just those who participated in the course. Second, participant learning measured through achievement among the various ACGME Clinician Educator Milestones could certainly have been influenced by self-assessment bias. We used recommendations from the Society of General Internal Medicine Education Committee to apply these milestones with faculty-guided self-assessment to mitigate this.^20^ In addition, the closer timing of the survey immediately before and after completion of the course, the respondents' commitment to self-assessment, resident reports of improvement in clinician-educator opportunity satisfaction, and their adoption of course material into their capstone projects all suggest that their changes in assessment were real. Third, while our outcomes reflect changes in resident satisfaction, learning of medical education concepts, and behavioral change as demonstrated through their implemented projects, we did not explore measures related to the highest tier of the Kirkpatrick model reflecting changes in organizational practice or patient outcomes. Last, while we demonstrated improvement in satisfaction with clinician-educator training in our residency program, opportunities still remain to push this further because nearly a third of the postimplementation cohort continues to be inadequately satisfied with opportunities for educator professional development.

Future directions of our program will involve collaboration with more faculty members within and external to our neurology department to ensure a culture of continuous improvement for the NCEP. Although the program was developed and piloted at a single institution, the framework used in the NCEP can feasibly be adopted by other neurology training programs interested in pushing clinician-educator professional development forward. From a resource allocation perspective, time commitment of faculty members involved in the program was relatively minimal with approximately 20 total hours of direct faculty involvement (3 1-hour sessions per week for the complete 6-week program, in addition to quarterly half-hour touch points), though 2 to 3-fold this time commitment was essential for indirect session planning and individual project review. With the growing enthusiasm surrounding medical education in neurology, we believe that most neurology residency programs can adapt the NCEP to suit their individual needs and resources. We have included in the supplementary materials the authors' original proposal to develop the NCEP to serve as a resource for other residency programs interested in reproducing a similar program (eAppendix 2).

In conclusion, we have developed and implemented a novel curriculum for neurology residents interested in becoming clinician-educators. The NCEP was well received by the participants and resulted in improved satisfaction with clinician-educator opportunities, increased learning of medical education concepts and skills, and behavioral change reflected in various capstone education projects. The NCEP provides a framework for other neurology residency programs to support and foster the development of future clinician-educators in neurology.

## Appendix Authors

**Table:**

Name	Location	Contribution
Rafid Mustafa, MD	Department of Neurology, Mayo Clinic, Rochester, MN	Drafting/revision of the manuscript for content, including medical writing for content; major role in the acquisition of data; study concept or design; analysis or interpretation of data
Sherri A. Braksick, MD	Department of Neurology, Mayo Clinic, Rochester, MN	Drafting/revision of the manuscript for content, including medical writing for content; study concept or design; analysis or interpretation of data
Jeremy K. Cutsforth-Gregory, MD	Department of Neurology, Mayo Clinic, Rochester, MN	Drafting/revision of the manuscript for content, including medical writing for content; analysis or interpretation of data
David B. Burkholder, MD	Department of Neurology, Mayo Clinic, Rochester, MN	Drafting/revision of the manuscript for content, including medical writing for content; study concept or design
Eoin P. Flanagan, MBBCh	Department of Neurology, Mayo Clinic, Rochester, MN	Drafting/revision of the manuscript for content, including medical writing for content; analysis or interpretation of data
Jonathan Graff-Radford, MD	Department of Neurology, Mayo Clinic, Rochester, MN	Drafting/revision of the manuscript for content, including medical writing for content; analysis or interpretation of data
Christopher J. Boes, MD	Department of Neurology, Mayo Clinic, Rochester, MN	Drafting/revision of the manuscript for content, including medical writing for content; analysis or interpretation of data
Andrea N. Leep Hunderfund, MD, MHPE	Department of Neurology, Mayo Clinic, Rochester, MN	Drafting/revision of the manuscript for content, including medical writing for content
Lyell K. Jones, MD	Department of Neurology, Mayo Clinic, Rochester, MN	Drafting/revision of the manuscript for content, including medical writing for content; study concept or design; analysis or interpretation of data
Elizabeth A. Coon, MD	Department of Neurology, Mayo Clinic, Rochester, MN	Drafting/revision of the manuscript for content, including medical writing for content; study concept or design; analysis or interpretation of data

## Glossary

ACGME: Accreditation Council for Graduate Medical Education
HMI: Harvard Macy Institute
NCEP: Neurology Clinician-Educator Program
PGY: postgraduation year
